# An Optimized Air-Core Coil Sensor with a Magnetic Flux Compensation Structure Suitable to the Helicopter TEM System

**DOI:** 10.3390/s16040508

**Published:** 2016-04-12

**Authors:** Chen Chen, Fei Liu, Jun Lin, Kaiguang Zhu, Yanzhang Wang

**Affiliations:** 1 Key Laboratory of Geo-exploration Instruments, Ministry of Education of China, Changchun 130061, China; cchen@jlu.edu.cn (C.C.); feiliu14@mails.jlu.edu.cn (F.L.); lin_jun@jlu.edu.cn (J.L.); zhukaiguang@jlu.edu.cn (K.Z.); 2 College of Instrumentation and Electrical Engineering, Jilin University, Changchun 130061, China

**Keywords:** helicopter TEM system, ACS, magnetic flux compensation structure, optimization of the ACS

## Abstract

The air-core coil sensor (ACS) is widely used as a transducer to measure the variation in magnetic fields of a helicopter transient electromagnetic (TEM) system. A high periodic emitting current induces the magnetic field signal of the underground medium. However, such current also generates a high primary field signal that can affect the received signal of the ACS and even damage the receiver. To increase the dynamic range of the received signal and to protect the receiver when emitting current rises/falls, the combination of ACS with magnetic flux compensation structure (bucking coil) is necessary. Moreover, the optimized ACS, which is composed of an air-core coil and a differential pre-amplifier circuit, must be investigated to meet the requirements of the helicopter TEM system suited to rapid surveying for shallow buried metal mine in rough topography. Accordingly, two ACSs are fabricated in this study, and their performance is verified and compared inside a magnetic shielding room. Using the designed ACSs, field experiments are conducted in Baoqing County. The field experimental data show that the primary field response can be compensated when the bucking coil is placed at an appropriate point in the range of allowed shift distance beyond the center of the transmitting coil and that the damage to the receiver induced by the over-statured signal can be solved. In conclusion, a more suitable ACS is adopted and is shown to have better performance, with a mass of 2.5 kg, resultant effective area of 11.6 m^2^ (*i.e.*, diameter of 0.496 m), 3 dB bandwidth of 66 kHz, signal-to-noise ratio of 4 (*i.e.*, varying magnetic field strength of 0.2 nT/s), and normalized equivalent input noise of 3.62 nV/m^2^.

## 1. Introduction

The helicopter transient electromagnetic (TEM) system has been widely used in the field of exploration over the last decade. A number of helicopter TEM systems have been developed, including AeroTEM, SkyTEM, HoisTEM, VTEM, and others [1,2,3,4]. A helicopter TEM system is composed of a transmitter and receiver; the former is utilized to emit a large bipolar periodic current that can induce the magnetic field signal of the underground medium, and the latter obtains the signal based on Faraday′s law and then stores the received data [5,6]. The air-core coil sensor (ACS) is the most important component of the receiver to obtain the magnetic field signal [7,8]. To achieve a superior detection performance of the helicopter TEM system suited to rapid surveying for shallow buried metal mine in rough topography, many stringent requirements for ACS must be met. First, the minimum signal-to-noise ratio (SNR) must meet the given requirement to reduce the deviation of the detection depth caused by ACS [9]. Second, the bandwidth of the ACS should be no less than 50 kHz, which can provide sufficient bandwidth to accurately characterize the shallow part of the crust [10]. Third, the signal acquired by the ACS is needed to avoid the interference from the helicopter in long distance transmissions [11]. Fourth, the selected material to fabricate the frame of the air-core coil should not exhibit magnetism to avoid magnetic interference. In addition, the mass of the ACS should be determined according to the actual situation to maintain the balance of the entire suspension frame. Finally, the induced signal will be larger than the allowable maximum value of the pre-amplifier if no bucking coil is used when the emitting current rises/falls. Electrical damage may occur in the pre-amplifier circuit of the receiver when the pre-amplifier works under an over-saturation state. To prevent damage to the receiver and increase the dynamic range of the secondary field while the emitting current turns off, a magnetic flux compensation structure must be designed [3,12,13]. Thus, the customized design and fabrication of an ACS with optimized performance is necessary.

Currently, many commercial ACSs are applied in helicopter TEM systems, such as the MTEM-AL MulTEM Loop and 3D-3LF sensor. The MTEM-AL MulTEM Loop sensor designed by Phoenix Geophysics (Canada) has excellent performance in terms of SNR, with an equivalent area of 100 m^2^ and a diameter of 1.1 m [13]. However, the matched magnetic flux compensation structure should be oversized for it to be applicable in the helicopter TEM system. The 3D-3LF sensor with the size of 0.6 × 0.6 × 0.2 m^3^ and a differential structure to transfer signals in long distance can meet the majority of requirements in helicopter TEM systems. Nevertheless, the 3 dB bandwidth of the 3D-3LF sensor is only 28 kHz, which is too narrow for mentioned helicopter TEM systems [14]. As a consequence, an optimized differential ACS with the required SNR, 3 dB bandwidth, and magnetic flux compensation structure to compensate for the above shortcomings should be developed for mentioned helicopter TEM systems.

This manuscript: (1) Analyzes the theory of magnetic flux compensation of the helicopter TEM system; (2) designs a magnetic flux compensation structure (bucking coil) suitable to helicopter TEM systems to compensate for the primary field when the emitting current rises/falls; (3) presents an ACS model with a differential structure to reduce the common-mode noise induced in cables during long distance transmission; (4) investigates the optimization of ACS in terms of size, 3 dB bandwidth, SNR, and normalized equivalent input noise; and (5) presents field experiment data to verify the performance of two optimized ACSs with bucking coil.

The rest of this paper is structured as follows: Section 2 presents the ACS matched with a bucking coil suitable to the helicopter TEM system. Section 3 illustrates the optimization of ACS based on the theory proposed in Section 2. In Section 4, two physical ACSs are fabricated according to the aforementioned optimized method. To verify the designed ACS, Section 5 and Section 6 describe the performances of two ACSs, which are conducted in a magnetic shielding room and in Baoqing County, China, respectively. The final section provides the conclusions and recommends directions for future research.

## 2. ACS Matched with Bucking Coil

**Figure 1:**
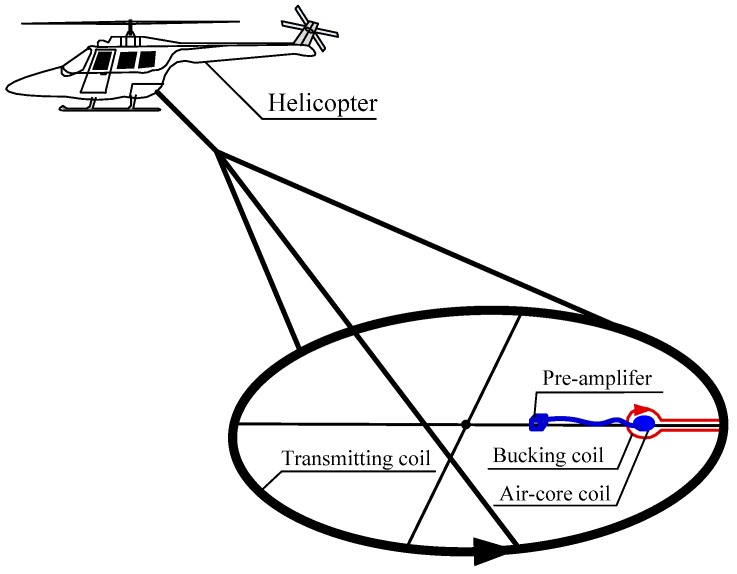
Helicopter transient electromagnetic (TEM) system configuration.

The configuration of the helicopter TEM system consists of a transmitting coil, bucking coil, and ACS, which is composed of an air-core coil and pre-amplifier, as shown in Figure 1.

A transmitting coil with a large diameter emits a bipolar trapezoidal wave to stimulate the magnetic field of the underground medium. The bucking coil is coincident-coplanar in geometry with the transmitting coil. In addition, the current in the bucking and transmitting coils is reversed to fulfill the magnetic flux compensation for ACS. Moreover, the ACS should be placed in the middle of the bucking coil. The air-core coil receives the magnetic field signal of the underground medium based on Faraday′s law. Meanwhile, the magnetic field signal is amplified by the differential pre-amplifier and is transmitted in long distance at a high common-mode rejection ratio.

### 2.1. Analysis of the Eccentric Bucking Coil

**Figure 2:**
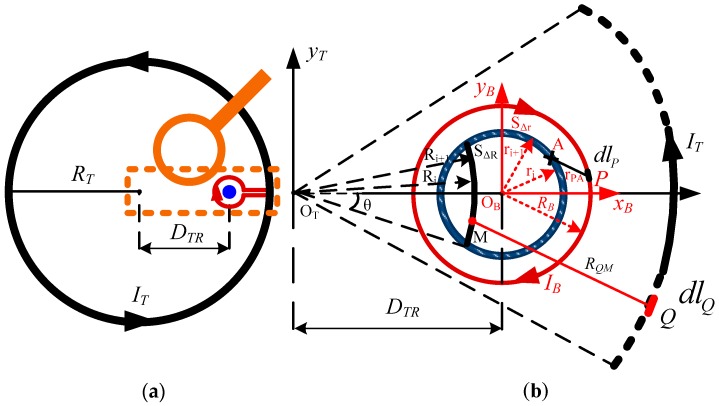
Top view of the suspension bracket and the magnetic field intensity. (**a**) Top view of the suspension bracket; (**b**) The magnetic field intensity of the suspension bracket.

The top view of the suspension bracket is shown in Figure 2a. The transmitting coil (black circle) and the bucking coil (red circle) are coincident-coplanar in geometry with the reversed currents *I_T_* and *I_B_*. These coils have *N_T_* and *N_B_* turns with the radii *R_T_* and *R_B_*, respectively. The transmitting coil can generate a large primary magnetic field when the emitting current rises/falls, which overwhelms the available magnetic field signal or even destroys the receiver. The bucking coil is utilized to cancel the large primary magnetic field based on the theory of magnetic flux compensation.

As seen in Figure 2b, according to the right-hand rule, the magnetic field intensity *B_M_* generated by the transmitting current points out of the plane. Correspondingly, the magnetic field intensity *B_A_* generated by the current in the bucking coil points into the plane. The total magnetic flux of the bucking coil inside can be obtained by the difference between the aforementioned two magnetic field intensities.

First, analyzing the magnetic field intensity at point M *B_M_*, the z-axis between the transmitting and bucking coils is not considered to be geometrically coincident-coplanar. Thus, the rectangular coordinate system x_T_, y_T_ is established in Figure 2b. At point M, the magnetic field intensity *B_M_* can be obtained as

(1) $$B_{M}(x_{T},y_{T})=N_{T}\cdot \frac{\mu_{0}}{4\pi }\cdot I_{T}\cdot \oint_{CT}\frac{dl_{Q}\times R_{QM}(x_{T},y_{T})}{R_{QM}{}^{3}}$$

where $\mu_{0}=4\pi \cdot 10^{-7}\;N/A^{2}$ is the permeability of the vacuum, *l_Q_* is an infinitesimal segment at point Q, *R_QM_* (x_T_, y_T_) is a vector from Q to M with the length *R_QM_*, and *C_T_* is the path of the line integral of the transmitting coil, described as $x^{2}{}_{T}+y^{2}{}_{T}=R_{T}{}^{2}$.

The magnetic flux $\phi_{BS_{\Delta R}}$ of the infinitesimal area is only a part of the magnetic flux of the infinitesimal annulus $S_{\Delta R}$, which can be calculated as

(2) $$\begin{array}{l} \phi_{BS_{\Delta R}}=B_{M}\cdot k\cdot S_{\Delta R}=B_{M}\cdot k\cdot \pi (R_{i+1}{}^{2}-R_{i}{}^{2})\\ \;=\frac{\mu_{0}\cdot N_{T}\cdot I_{T}\cdot k\cdot (R_{i+1}{}^{2}-R_{i}{}^{2})}{4}\cdot \oint_{C_{T}}\frac{dl_{Q}\times R_{QA}(x_{T},y_{T})}{r_{QM}{}^{3}} \end{array}$$

where $k=\frac{\theta }{\pi }$.

The magnetic flux $\phi_{Tr}$ caused by the transmitting coil through the circle with the radius *r* can be calculated as

(3) $$\phi_{Tr}=\frac{\mu_{0}\cdot N_{T}\cdot I_{T}}{2}\cdot \int_{D_{TR}-r}^{D_{TR}+r}k\cdot R\text{​​}\cdot \;\oint_{C_{T}}\frac{R_{QM}(x_{T},y_{T})\times dl_{Q}}{R_{QM}{}^{3}}dR$$

Second, with respect to the magnetic field intensity *B_A_*, another coordinate x_B_, y_B_ is established. The value of *B_A_* can be calculated by Biot Savart law, as shown in Equation (4):

(4) $$B_{A}(x_{B},y_{B})=N_{B}\cdot \frac{\mu_{0}}{4\pi }\cdot I_{B}\cdot \oint_{C_{B}}\frac{dl_{P}\times r_{PA}(x_{B},y_{B})}{r_{PA}{}^{3}}$$

where *l_P_* is an infinitesimal segment at point P, $r_{PA}(x_{B},y_{B})$ is a vector from P to A with the length $r_{PA}$, and *C_B_* is the path of the line integral of the bucking coil , described as $x^{2}{}_{B}+y^{2}{}_{B}=R_{B}{}^{2}$.

The value of the magnetic field intensity at the circle ring of $r_{i}$ is equal to *B_A_* because of its geometric symmetry. Thus, the magnetic flux $\phi_{BS_{\Delta r}}$ through the infinitesimal annulus $S_{\Delta r}$ can be computed as

(5) $$\phi_{BS_{\Delta r}}=B_{A}\cdot S_{\Delta r}=B_{A}\cdot \pi (r_{i+1}{}^{2}-r_{i}{}^{2})=\frac{\mu_{0}\cdot N_{B}\cdot I_{B}\cdot (r_{i+1}{}^{2}-r_{i}{}^{2})}{4}\cdot \oint_{C_{B}}\frac{dl_{P}\times r_{PA}(x_{B},y_{B})}{r_{PA}{}^{3}}$$

The magnetic flux $\phi_{Br}$ of the circle area with the radius *r* is the sum of $\phi_{BS_{\Delta r}}$, which can be calculated as

(6) $$\phi_{Br}=\frac{\mu_{0}\cdot N_{B}\cdot I_{B}}{2}\cdot \int_{0}^{r}r\cdot \oint_{C_{B}}\frac{r_{PA}(x_{B},y_{B})\times dl_{P}}{r_{PA}{}^{3}}dr$$

As a consequence, the total magnetic flux $\phi_{r}$ through the circle with the radius r can be described as

(7) $$\begin{array}{l} \phi_{r}=\phi_{Br}-\phi_{Tr}\\ \;=\frac{\mu_{0}\cdot N_{B}\cdot I_{B}}{2}\cdot \int_{0}^{r}r\cdot \oint_{C_{B}}\frac{r_{PA}(x_{B},y_{B})\times dl_{P}}{r_{PA}{}^{3}}dr-\frac{\mu_{0}\cdot N_{T}\cdot I_{T}}{2}\cdot \int_{D_{TR}-r}^{D_{TR}+r}k\cdot R\text{​​}\cdot \;\oint_{C_{T}}\frac{R_{QM}(x_{T},y_{T})\times dl_{Q}}{R_{QM}{}^{3}}dR \end{array}$$

Hence, the bucking coil with the reversed current can compensate for the magnetic flux completely under certain specifications. The total magnetic field of ACS is greatly neutralized, and the ACS has better performance and reliability in geophysical exploration.

In addition, an approximation method to calculate *Φ_Tr_* is available if the area of the air-core coil is considerably smaller than that of transmitter loop. The magnetic field intensity *B_M_*(*x_T_*,*y_T_*) in the area of radius *r* is considered as a constant value *B_OB_*, which is the magnetic field intensity at the center point. Thus, the magnetic flux *Φ_Tr_* generated by the transmitting current can be calculated as $\phi_{Tr}\approx B_{\text{OB}}\cdot \pi \cdot r^{2}$, which can replace Equation (3). The error between the approximation result and the accurate result depends on the area ratio of the receiver to the transmitter loop.

### 2.2. Equivalent Electrical Model of an Air-core Coil

**Figure 3:**
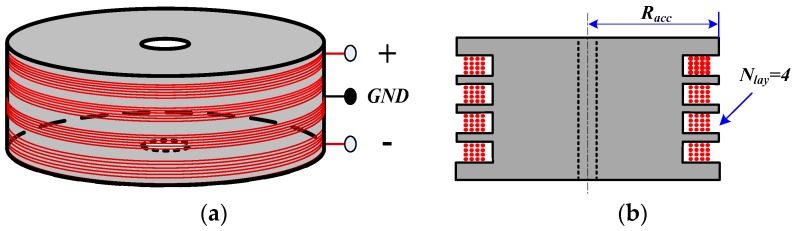
(**a**) An air-core coil with a differential structure; and (**b**) its typical design (*R_acc_*—radius of the air-core coil, *N_lay_*—number of layers).

An air-core coil with a differential structure is designed to reduce the common-mode noise (Figure 3). As seen from Figure 3, several turns of wire are wound on a nonmagnetic circular framework with four sections, which can decrease the distributed capacitance.

The air-core coil is used to measure the variation of the magnetic field based on Faraday′s law.

(8) $$V=-n\cdot \frac{d\phi }{dt}=-n\cdot A\cdot \frac{dB}{dt}=-n\cdot \pi \cdot R_{acc}{}^{2}\cdot \frac{dB}{dt}$$

where *Φ* is the magnetic flux through the air-core coil, *A* and *R_acc_* are the area and radius of the air-core coil, respectively, and n is the turns of wire.

**Figure 4:**
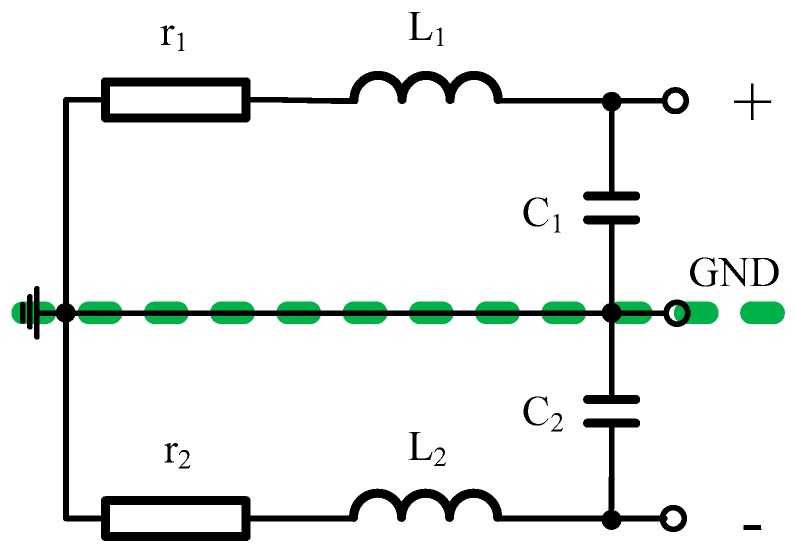
Electrical model of the air-core coil.

The electrical model of the air-core coil is composed of the resistor, inductor, and capacitor, as shown in Figure 4, where *r*, *L*, and *C* are the resistance, inductance and capacitance of the air-core coil, respectively.

The self-thermal noise of the air-core coil is the only factor that impacts its resolution according to the electrical model. Its self-thermal noise *V_t_* is generated by the resistance and is affected by the temperature as well.

(9) $$V_{t}=\sqrt{4\cdot k_{B}\cdot T\cdot r}=\sqrt{4\cdot k_{B}\cdot T\cdot \rho_{r}\cdot \frac{n\cdot \pi \cdot 2\cdot R_{acc}}{\pi \cdot {\left(\frac{d}{2}\right)}^{2}}}=\frac{\sqrt{32\cdot k_{B}\cdot T\cdot \rho_{r}\cdot n\cdot R_{acc}}}{d}$$

where the Boltzman factor $k_{B}=1.38\cdot 10^{-23}W\cdot s/K$, *T* is Kelvin temperature, $\rho_{r}$ is the electrical resistivity of the wire, and *d* is the diameter of the wire.

Thus, the SNR of the air-core coil is calculated as

(10) $$SNR=\frac{V}{V_{t}}=-\frac{\pi \cdot d}{\sqrt{32\cdot k_{B}\cdot T\cdot \rho_{r}}}\cdot \sqrt{n}\cdot R_{acc}{}^{\frac{3}{2}}\cdot \frac{dB}{dt}$$

It can be derived from Equation (10) that the SNR of the air-core coil is proportional to the turns of wire with half power and radius *R_acc_* the power of 1.5. Considering the requirement of magnetic flux compensation and the effect of the self-noise of ACS on the deviation in detection depth, the *R_acc_* and *n* of the air-core coil should be limited within a certain value. The requirement details are discussed in Section 3.1 and Section 3.2. In addition, the original value of *V* produced by the air-core coil cannot be transmitted in long distance so that a suitable pre-amplifier is necessary. The mentioned electrical parameters of the air-core coil, such as self-resistance, inductance and capacitance, are the key factors that can determine the selection of the operational amplifier for the pre-amplifier circuit.

The rotation of the sensor in Earth′s magnetic field can cause motion noise within a wide frequency range while flying. This section aims to analyze the electrical noise of the air-core coil. Thus, the motion noise is regarded as another type of noise and not considered when calculating the parameter SNR.

### 2.3. Equivalent Schematic of ACS with a Differential Pre-Amplifier Circuit

**Figure 5:**
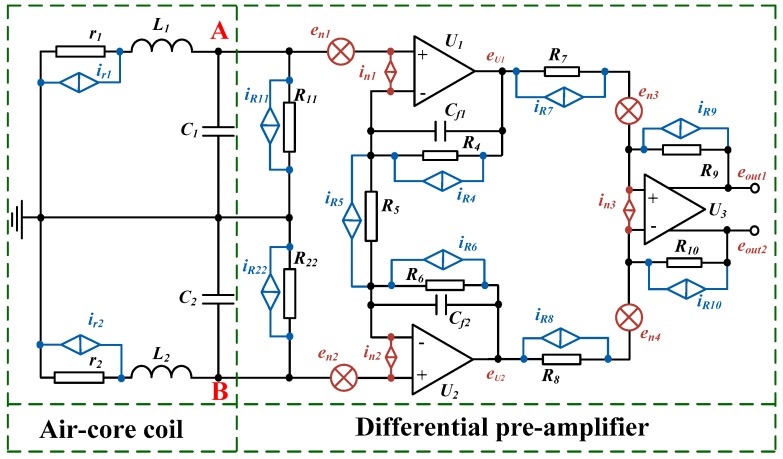
Equivalent schematic of ACS with pre-amplifier circuit.

The signal produced by the air-core coil is too low to transmit in long distance. Thus, an equivalent schematic of an ACS with a pre-amplifier circuit is suitable to the air-core coil, as shown in Figure 5 [15,16,17]. The pre-amplifier circuit is connected to the air-core coil with a differential structure to decrease the common-mode noise. Moreover, the equivalent input noise of ACS is required to meet the requirement of the mentioned helicopter TEM system.

As seen in Figure 5, the integrated operational amplifiers U_1_, U_2_, and U_3_ connected with the resistors R_4_, R_5_, R_6_, R_7_, R_8_, R_9_, and R_10_ can regulate an appropriate gain to amplify the low-voltage signal produced by the air-core coil in the order of a few mV to the operational range for further processing in several voltages. R_11_ and R_22_ are matched resistors that adjust the working state of the air-core coil in accordance with the requirement of the mentioned helicopter TEM system.

The thermal noise of all the resistors and the input voltage noise and input current noise of the integrated operational amplifiers U_1_, U_2_ and U_3_ are all illustrated in Figure 5. The thermal noise of the resistor can be described as $i_{R}=\sqrt{4k_{B}TR}$, where *R* is the resistance of the resistor. The *e_n1_*, *e_n2_*, and *e_n3_* and *i_n1_*, *i_n2_* and *i_n3_* are the input voltage noise and input current noise, respectively, of the integrated operational amplifiers U_1_, U_2_, and U_3_. The equivalent input noise of ACS at point A or B can then be calculated as

(11) $$\left|V_{An}\right|=\left|V_{Bn}\right|=\sqrt{V_{An}^{2}+V_{Bn}^{2}}\;=\sqrt{V_{en}^{2}+V_{in}^{2}+V_{Rn}^{2}}$$

|VAn|=|VBn|=Vn2 given the symmetrically differential structure, and *V_n_* is the total equivalent input noise, and *V_en_* is the sum of the voltage noises *e_n1_*, *e_n2_*, and *e_n3_*. *V_in_* is the sum of the current noises *i_n1_*, *i_n2_*, and *i_n3_*. *V_Rn_* is the total thermal noise of all the resistors.

Analyzing point A as an example, the *V_en_* generated by *e_n1_* and *e_n3_* is expressed as

(12) $$V_{en}=\sqrt{{\left(e_{n1}\right)}^{2}+{\left(\frac{e_{n3}}{G_{1}}\right)}^{2}}\;$$

where *G_1_* is the gain of the integrated operational amplifier U_1_.

The input current noise *V_in_* produced by *i_n1_* and *i_n3_* can be described as

(13) $$V_{in}=\sqrt{{\left(i_{n1}Z\right)}^{2}+{\left(i_{n1}R_{eq1}\right)}^{2}+{\left(\frac{i_{n3}R_{eq2}}{G_{1}}\right)}^{2}}\;$$

where *Z*, *R_eq1_*, and *R_eq2_* can be expressed as

(14) $$Z=\left(r_{1}+j\omega L_{1}\right)||\frac{1}{j\omega C_{1}}||R_{11}=\frac{r_{1}+j\omega L_{1}}{1-\omega^{2}L_{1}C_{1}+\frac{r_{1}}{R_{11}}+j\omega (C_{1}r_{1}+\frac{L_{1}}{R_{11}})}$$

where ω is the angular frequency, ω = 2πf. Furthermore,

(15) $$R_{eq1}=R_{4}||\frac{R_{5}}{2}=\frac{R_{4}R_{5}}{R_{5}+2R_{4}}$$

(16) $$R_{eq2}=R_{7}||R_{9}=\frac{R_{7}R_{9}}{R_{7}+R_{9}}$$

Finally, the thermal noise *V_Rn_* yielded by all the resistors can be obtained as

(17) $$\begin{array}{l} V_{Rn}=\sqrt{{\left(i_{r1}Z\right)}^{2}+{\left(i_{R11}Z\right)}^{2}+{\left(i_{R4}R_{eq1}\right)}^{2}+{\left(i_{R5}R_{eq1}\right)}^{2}+{\left(\frac{i_{R7}R_{eq2}}{G_{1}}\right)}^{2}+{\left(\frac{i_{R9}R_{eq2}}{G_{1}}\right)}^{2}}\;\\ \;=\sqrt{4kT\left(\frac{r_{1}R_{11}Z^{2}}{r_{1}+R_{11}}+\frac{\left(R_{4}+R_{5}\right)R_{eq1}{}^{2}}{R_{4}R_{5}}+\frac{\left(R_{7}+R_{9}\right)R_{eq2}{}^{2}}{R_{7}R_{9}G_{1}^{2}}\right)} \end{array}$$

The total equivalent input noise *V_n_* can be calculated as $V_{n}=\sqrt{2}\cdot V_{An}=\sqrt{2}\cdot V_{Bn}$, according to the complete symmetrical structure of the pre-amplifier circuit. Thus, the noise contributions from each component can guide the optimization of the circuit performance.

In summary, the above analysis presents all these aspects. The magnetic flux compensation structure (bucking coil) is designed to create a zero-primary field area for ACS. In addition, the specifications of the air-core coil and the noise sources of ACS both significantly impact the exploration results of the helicopter TEM system. Thus, the optimization of the aforementioned aspects of ACS is of great importance.

## 3. Specifications for the Optimization of the ACS

### 3.1. Optimization of the Geometric Location of the Bucking Coil

To avoid covering the available magnetic field signal or even destroying the receiver, the radius of the air-core coil must meet the helicopter TEM system′s requirement that the bucking coil can compensate for the magnetic flux completely, *Φ_r_* = 0. A helicopter TEM suspension bracket is given with a transmitting coil (*i.e.*, radius *R_T_* = 6 m and turns *N_T_* = 5) and a bucking coil (*i.e.*, radius *R_B_* = 0.6 m and turns *N_B_* = 1). The bucking coil can be shifted at a distance (*D_TR_*) from 4.62 m to 4.7 m beyond the center of the transmitting coil.

**Figure 6:**
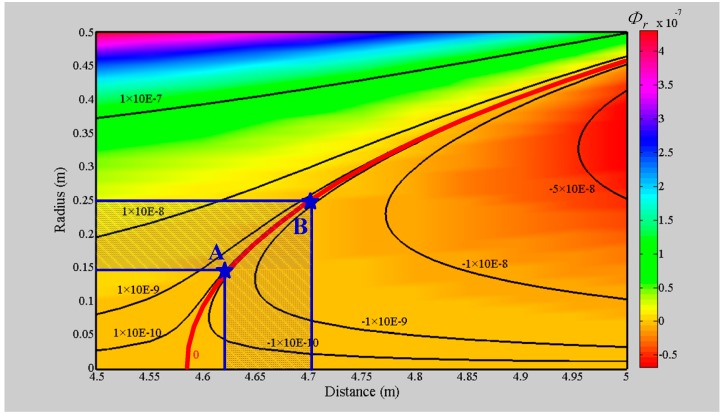
Contour map of the magnetic flux *Φ_r_ versus* *D_TR_* and the circle radius *R_acc_*.

Thus, the magnetic flux *Φ_r_* inside the bucking coil is computed according to Equation (7) by changing *D_TR_*, as shown in Figure 6.

In Figure 6, the value of *Φ_r_* inside the bucking coil can be separated into two components, where one is *Φ_r_* > 0 to illustrate that the magnetic flux caused by the bucking coil overwhelms the magnetic flux induced by the transmitting coil, and the other is reversed. The red line represents that bucking coil compensating for the magnetic flux exactly, where *Φ_r_* = 0. In consideration of the actual requirement of *D_TR_*, only the values from points A to B are available. As a consequence, the radius *R_acc_* of the air-core coil can be determined as

(18) $$subject\;to\;R_{acc}\in \{\text{ valu}es\;\phi_{r}=0,\;4.62\;m\leq D_{TR}\leq 4.7\;m\}$$

where 0.15 m ≤ *R_acc_* ≤ 0.25 m according to Figure 6.

### 3.2. Geometric Optimization of the Air-Core Coil

The impact of the self-noise of ACS on the deviation of the detection depth should not exceed 2% when the half-space earth electrical conductivity is set at 0.01 S/m. Accordingly, the SNR of the air-core coil should be larger than 4 when the varying magnetic field is 0.2 nT/s. Thus, the optimum values of n and Racc according to Equation (10) are as follows:

(19) $$subject\;to\;SNR(n,R_{acc})\geq 4，when\;\frac{dB}{dt}=0.2\;nT/s\;$$

**Figure 7:**
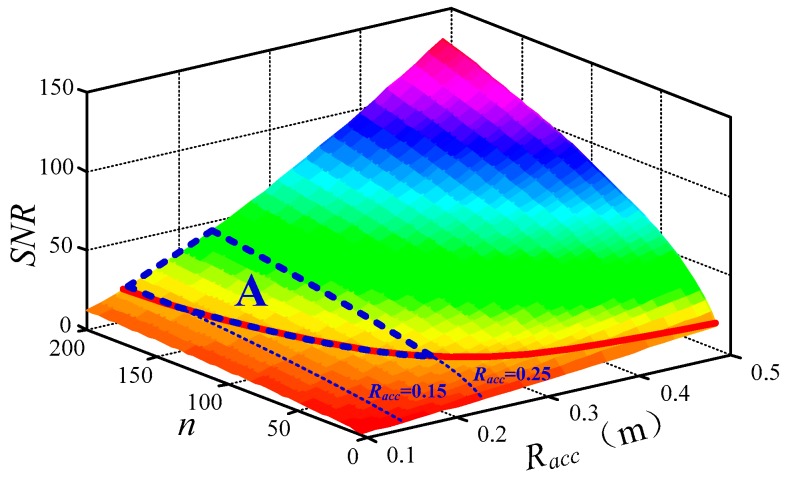
Contour map of signal-to-noise ratio (SNR) *versus* *R_acc_* and *n.*

A contour map of the SNR of the air-core coil, which depends on n and *R_acc_*, is shown in Figure 7.

As can be seen in Figure 7, the red line is an equipotential line where SNR = 4. The values above the equipotential line are larger than 4, whereas the values below are smaller than 4. Certainly, the values in the upper area are available. In consideration of the preceding discussion on the magnetic flux compensation of the bucking coil, there is an adaptive region A for *R_acc_* from 0.15 m to 0.25 m, which is marked in the above figure. Thus, the values of n and *R_acc_* in region A are the matched parameters of the air-core coil suited to the helicopter TEM system.

Moreover, the output voltage induced from ACS should not be less than 1430 nV because the receiver has a 24-bit analog-to-digital converter (ADC) with the voltage range of ±12 V. When the magnetic field varies at 0.2 nT/s, and the amplification factor is 620, the resultant equivalent area S of the air-core coil should be larger than 11.6 m^2^. The contour blue line is denoted by S = 11.6 m^2^, as shown in following figure, which is a two-dimensional view of Figure 7.

**Figure 8:**
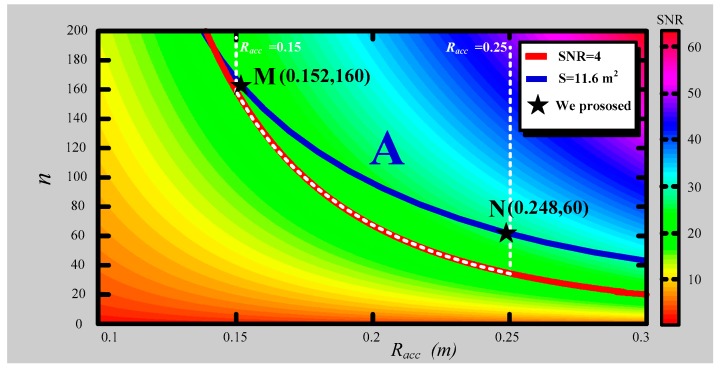
Two-dimensional image of Figure 7.

In Figure 8, the segment of the blue line from M to N, *S* = 11.6 m^2^, is in the available region A. At point M, the air-core coil has the radius *R_acc_* = 0.152 m, and *R_acc_* = 0.248 m at point N. The chosen *R_acc_* = 0.152 m is a little larger than the minimum available value 0.15 m, and the selected *R_acc_* = 0.248 is a little less than 0.25 m with number of turns 160 and 60, which are both multiples of 4. Notably, points M and N are the extreme conditions, which meet the requirements of the helicopter TEM system.

According to the preceding discussion, the air-core coil with the minimum *R_acc_* = 0.152 m, turns *n* = 160, and maximum *R_acc_* = 0.248 m, *n* = 60 can fulfill the magnetic flux compensation exactly. Comparative experiments are conducted in the latter part of the section to demonstrate the difference in their performances.

### 3.3. Optimization of the Pre-Amplifier Circuit

**Figure 9:**
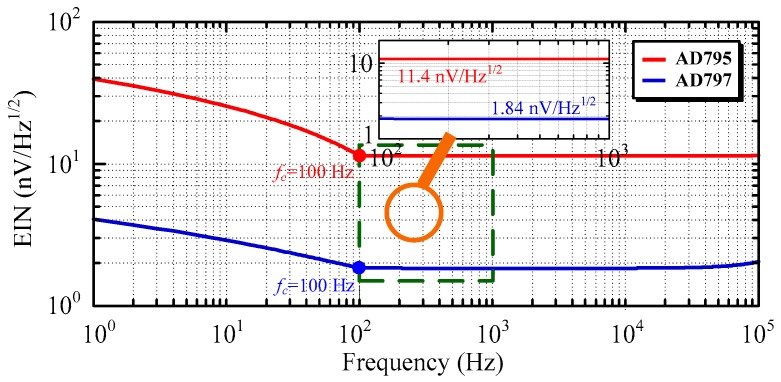
Equivalent input noise calculated using AD797 and AD745.

To decrease the equivalent input noise of the ACS, the low-noise pre-amplifier circuit is required. Two kinds of operational amplifiers are utilized for comparison, the lowest-voltage noise operational amplifier and the lowest-current noise operational amplifier among the commercial operational amplifiers. Correspondingly, the lowest-voltage noise operational amplifier AD797 (Analog Devices company) and the lowest-current noise operational amplifier AD795 (Analog Devices company) are adopted, where AD797 has the input voltage noise $e_{n}=0.9\text{ nV/}\sqrt{\text{Hz}}$ and input current noise $i_{n}=2.0\text{ pA/}\sqrt{\text{Hz}}$, and AD795 has $e_{n}=11\text{ nV/}\sqrt{\text{Hz}}$ and $i_{n}=0.6\text{ fA/}\sqrt{\text{Hz}}$. Moreover, the resistors used to regulate the gain of the pre-amplifier circuit, especially the resistors R_4_, R_5_, and R_6_, should not have a high resistance to reduce the thermal noise. The simulation of the equivalent input noise (EIN) of the ACS using the component parameters in Section 4.2 is demonstrated in Figure 9.

Obviously, the ACS using AD797 has a lower equivalent input noise than that using AD795. Both of them have 1/f noise when the frequency is lower than the corner frequency, 100 Hz. At the frequency bandwidth from 100 Hz to 100 kHz, the equivalent input noise of ACS using AD797 is smaller than that using AD795, which are $1.84\text{ nV/}\sqrt{\text{Hz}}$ and $11.4\text{ nV/}\sqrt{\text{Hz}}$, respectively. Given the small distribution parameters of the air-core coil (*i.e.*, resistance, inductance, and capacitance), their contribution to the equivalent input noise can be neglected when the frequency is lower than 10 kHz. However, the equivalent input noise slightly rises, along with an increase in the frequency larger than 10 kHz, which indicates that the distribution parameters of air-core coil can affect the specification of ACS.

**Figure 10:**
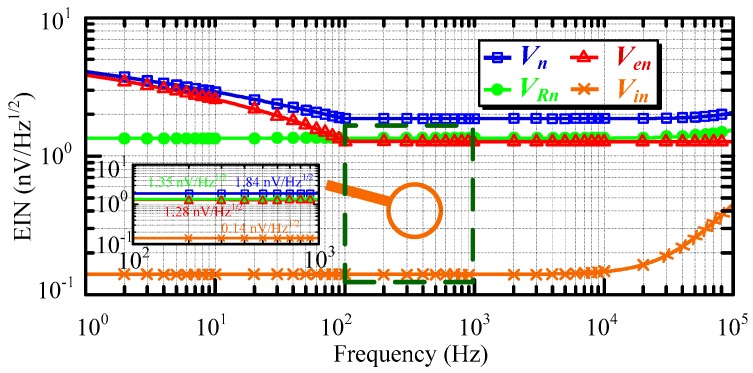
Noise sources of air-core coil sensor (ACS) using AD797.

To investigate the noise contributions from the three sources, the input voltage noise, input current noise, thermal noise, and equivalent input noise of ACS using AD797 is disassembled in Figure 10.

The thermal noise *V_Rn_* of the pre-amplifier circuit is almost constant during the whole frequency bandwidth. The input voltage noise *V_en_* has a similar contribution as the thermal noise *V_Rn_*, apart from in 1/f noise region, where it is even larger than the thermal noise *V_Rn_*. Moreover, the input current noise *V_in_* can be neglected when the frequency is lower than 10 kHz, and it rises with an increase in the frequency in the high-frequency bandwidth (>10 kHz). Thus, the thermal noise *V_Rn_* and input voltage noise *V_en_* are the two major factors that impact the total equivalent input noise *V_n_*. The low-voltage noise operational amplifier AD797 and low-resistance resistor are available approaches to reduce the input voltage noise and thermal noise in the mentioned helicopter TEM system.

From the above discussion, two ACSs with optimized physical and electrical specifications are designed to meet the special requirements of the magnetic flux compensation of the helicopter TEM system. The fabrication of the two ACSs is demonstrated below.

## 4. Fabrication of ACS

According to the requirements of the helicopter TEM system, two ACSs are fabricated in this section. The ACS is divided into two parts: the air-core coil and the differential pre-amplifier circuit, which are represented separately below.

### 4.1. Fabrication of the Air-Core Coil

**Figure 11:**
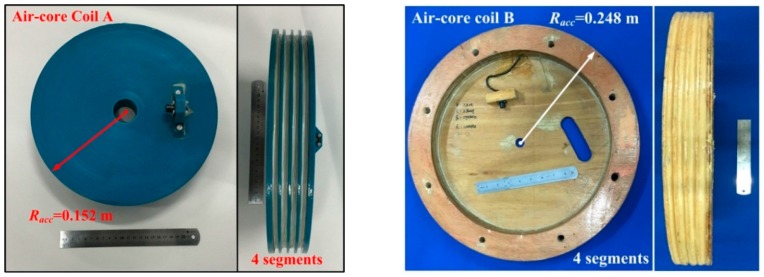
Two air-core coils.

The optimization methods discussed above are used to fabricate two air-core coils, shown in Figure 11. Their frame materials are nylon and wood without magnetism, air-core coils A and B, respectively.

The resultant effective area of air-core coil A (nylon material) is 11.6 m^2^, and its mass is 4.1 kg. By contrast, the resultant effective area and mass of air-core coil B (wood material) are 11.6 m^2^ and 2.5 kg, respectively. The detailed parameters of the two air-core coils are demonstrated in Table 1.

Both of the air-core coils use the same wire with four segments of frame structure. Air-core coil A has a larger resistance, capacitance, inductance, and lower response frequency given that it has more turns than air-core coil B.

### 4.2. Fabrication of the Pre-Amplifier Circuit

A differential pre-amplifier circuit matched with both the two air-core coils is designed. The components utilized in the pre-amplifier circuit are given in Table 2.

The selected components of the pre-amplifier circuit were carefully matched, which guarantees the differential structure. The resistance of R_11_ and R_22_ are chosen to make the step response of the air-core coil under the critical damping state. R_4_, R_5_, and R_6_ have low resistance in several hundred ohms to reduce the thermal noise. Given that R_7_, R_8_, R_9_ and R_10_ have a small contribution to the equivalent input noise, as shown in Equation (17), their resistance can be in kilo-ohms to increase the input impedance of the second stage of the pre-amplifier circuit. The gain of the pre-amplifier circuit G can be calculate as

(20) $$G=\left(1+2\frac{R_{4}}{R_{5}}\right)\cdot \frac{R_{9}}{R_{7}}$$

The electrical performances of ACSs, such as frequency response and equivalent input noise, are shown in Section 5.

## 5. Electrical Performances of ACSs

To compare the electrical performances of the fabricated ACSs, experiments are conducted in a magnetic shielding room. The experiments include the comparison of the frequency response and equivalent input noise of the ACSs. The results are demonstrated below.

### 5.1. Comparison of the Frequency Response of ACSs

**Figure 12:**
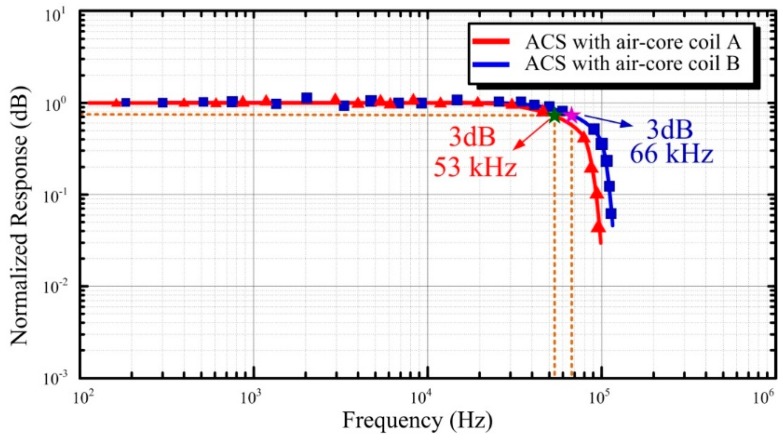
3 dB bandwidth of the two ACSs.

The 3 dB bandwidth of the ACS needs to meet the requirement of no less than 50 kHz in the helicopter TEM exploration. The 3 dB bandwidth performances of the two manufactured ACSs are verified in the shielding room, as shown in Figure 12.

In Figure 12, although both of the two ACSs are capable of meeting the 3 dB bandwidth requirement of the helicopter TEM exploration, the ACS with air-core coil A has a narrower 3 dB bandwidth of 53 kHz, whereas the ACS with air-core coil B has a wider 3 dB bandwidth of 66 kHz.

### 5.2. Comparison of the Equivalent Input Noise of ACSs

**Figure 13:**
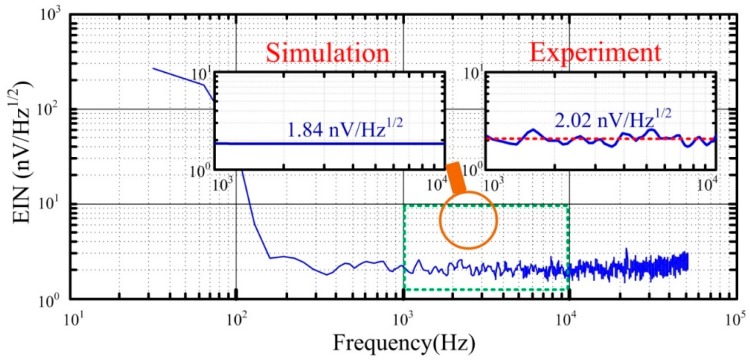
Equivalent input noise of ACS with air-core coil B.

The equivalent input noise of the ACSs is illustrated and simulated in Section 3. To verify the simulation results, the equivalent input noise of the ACSs is measured in the magnetic shielding room. Using ACS with air-core coil B as an example, the test result is compared with simulation result of the equivalent input noise, as shown in Figure 13.

Figure 13 illustrates that the test result of the equivalent input noise of ACS has a good coincidence with the simulation result. However, the test performance is approximately 2.02 nV/Hz^1/2^, which is slightly larger than the simulation result because of the influence of residual magnetism noise in the shielding room. The noise performance of ACS with air-core coil A is 2.86 nV/Hz^1/2^, which is higher than ACS with air-core coil B, given its larger electrical specifications (*i.e.*, resistance, capacitance, and inductance). The normalized equivalent input noise (*EIN_nor_*) of ACS can then be calculated as

(21) $$EIN_{nor}=\frac{\sqrt{\int_{B}EIN^{2}}}{S}$$

where *B* is the 3 dB bandwidth of the ACS and *S* is the resultant equivalent area of the air-core coil.

The values of the normalized equivalent input noise of ACS can be acquired, where the ACS with air-core coil B is 2.84 nV/m^2^ and that with air-core coil A is equal to 3.74 nV/m^2^.

The performance of the 3 dB bandwidth and equivalent input noise of ACSs both meet the requirements of the helicopter TEM exploration suited to rapid surveying for shallow buried metal mine in rough topography. The fabricated ACS with air-core coil B has a wider 3 dB bandwidth and lower equivalent input noise than that with air-core coil A. The results show that the ACS with air-core B is more suitable for application in the helicopter TEM exploration than that with air-core A. The comparison of field experiments using the two ACSs is performed in Section 6, which verifies the aforementioned conclusion.

## 6. Field Experiments in Baoqing County

**Figure 14:**
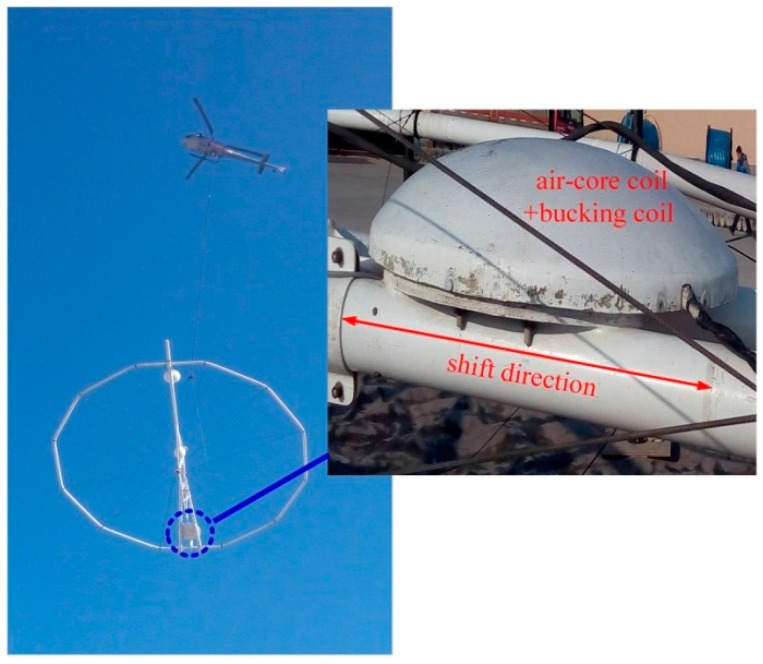
Helicopter TEM system and magnetic flux compensation structure.

Using the designed ACSs, which are the critical parts in the helicopter TEM system, field experiments were conducted in Baoqing County, Heilongjiang Province, China in November 2015. The helicopter TEM system and magnetic flux compensation structure are illustrated in Figure 14.

In Figure 14, the bucking coil can shift a distance of 0.08 m (4.62 m to 4.7 m) along the shift direction. The ground experiments are operated to verify the feasibility of using the magnetic flux compensation structure (bucking coil). Moreover, two flights of the helicopter TEM exploration with ACSs are conducted to compare the specifications of the two ACSs by estimating the received field data.

### 6.1. Ground Experiment Using the Magnetic Flux Compensation Structure

**Figure 15:**
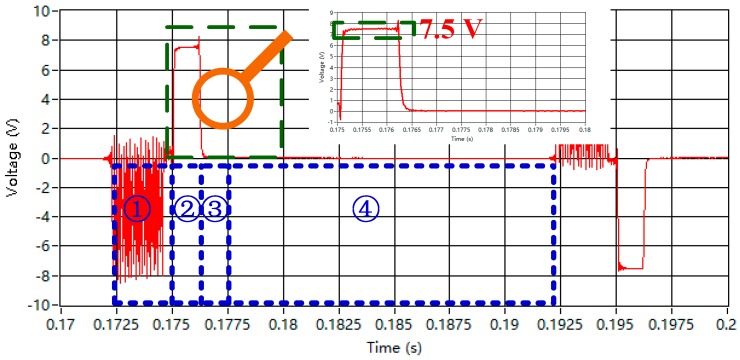
Waveform captured from the helicopter TEM system.

Air-core coil B is placed at the center of the bucking coil, and the bucking coil can shift at a distance (*D_TR_*) of only 0.08 m from 4.62 m to 4.7 m beyond the center of the transmitting coil. The waveform captured from the helicopter TEM system when the center of the bucking coil is shifted to the point at *D_TR_* = 4.685 m is demonstrated in Figure 15.

There are three periods of the received waveform. First, period ① indicates the time when the emitting current rises and stays constant. The receiving data are unavailable because they are merged into the signal induced by the modulated emitting current. In addition, period ② starts at the point when the emitting current begins to decline and stops at the time when the emitting current is cut off. The voltage is equal to 7.5 V, which is induced by the emitting current. The induced high voltage will decrease the dynamic range of signal or even destroy the receiver under some severe conditions. Finally, the waveform at period ③ is the magnetic field response of the underground medium when no emitting current exists.

**Figure 16:**
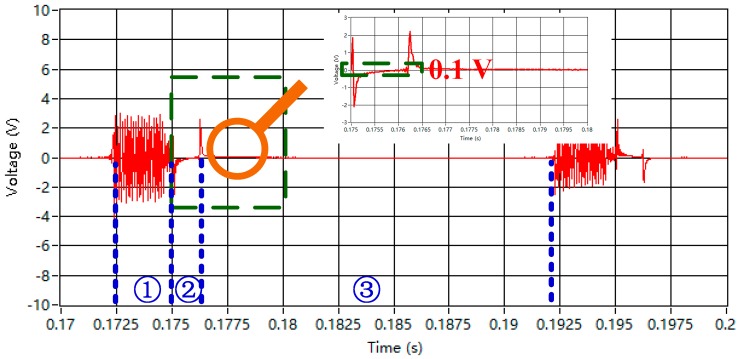
Waveform captured from the helicopter TEM system.

By contrast, the bucking coil is adjusted at the appropriate point, *D_TR_* = 4.693 m. The result of the received waveform is shown in Figure 16.

The primary field response caused by the emitting current can be compensated for nearly up to zero. Under the actual condition, the secondary field signal cannot be used for data interpretation because of the position deviation between the bucking coil and the transmitter loop when on the ground and during flight.

The comparison shows that the primary field response can be compensated when the bucking coil is placed at an appropriate point. This procedure results in the following benefits of using the bucking coil. First, the over-saturated induced signal can be reduced to protect the receiver. Second, the high magnification factor of the pre-amplifier can be utilized because of the reduction in the magnitude of the primary field signal. This reduction increases the dynamic range of the secondary field signal.

### 6.2. Field Experiments Using Two ACSs

Field experiments were conducted in Baoqing County. The suspension bracket is about 40 m above the ground, which is drawn by a helicopter with a fly speed of 40 knots. To compare the specifications of the two ACSs, the helicopter flight is conducted over the same survey line of about twice the 10 km. The normalization result of the received data is given below.

**Figure 17:**
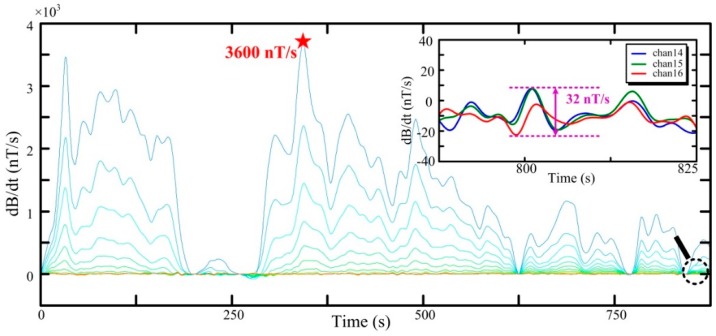
Normalization result of the received data with air-core coil B.

A total of 16 sampling channels in the normalization results are shown in Figure 17. The channels with a small number correspond with the data received earlier, which indicate the signal intensity of the underground medium. The large ones represent the data received later, especially the last three channels, which are deemed as the background noise.

**Figure 18:**
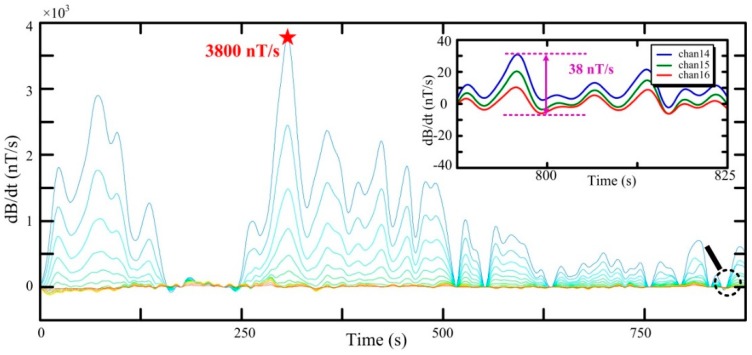
Flight line with air-core coil A.

The normalization results of received data using air-core coil B are as follows: maximum signal of 3600 nT/s, background noise of 32 nT/s, and ratio of maximum signal to background noise of 112.5. Comparatively, the normalization results using air-core coil A are given in Figure 18.

As can be seen from Figure 17 and Figure 18, the profiles of the two figures are similar and can be used to interpret information on the underground medium. The normalization results using air-core A are as follows: the larger maximum signal is 3800 nT/s, the larger background noise is 38 nT/s, and the ratio of the maximum signal to background noise is 100. In addition, air-core coil B with a larger SNR can obtain a higher-quality signal of the underground medium in helicopter TEM exploration.

From the above discussion, the magnetic flux compensation structure with ACS can compensate for the primary field response as expected, can increase the dynamic range, and protect the receiver in helicopter TEM exploration. Moreover, field experiments are performed using the two optimized ACSs that both meet the requirements of helicopter TEM exploration, and the ACS with a higher SNR provides a higher-quality signal. Thus, the ACS with a higher SNR incorporating the magnetic flux compensation structure is available for helicopter TEM exploration.

## 7. Conclusions and Prospects

For this study, ACSs combined with a magnetic flux compensation structure (bucking coil) are designed, fabricated, and tested to remove the primary field response when the emitting current rises/falls in helicopter TEM exploration. The physical and electrical models of the air-core coil are introduced, and its parameters meet the requirements of the helicopter TEM system suited to rapid surveying for shallow buried metal mine in rough topography. A differential pre-amplifier circuit suitable to the air-core coil transfers the signal in a long distance. The two ACSs are fabricated with optimized electrical performances, including the resultant effective area, 3 dB bandwidth, SNR, and normalized equivalent input noise, which are tested in a magnetic shielding room. Moreover, the field experiment data implemented in Baoqing Country show that the primary field response can be compensated for absolutely when the bucking coil is placed at an appropriate point, and the damage to the receiver induced by the over-statured signal can be removed. Eventually, a more suitable ACS is adopted and is shown to have better performance, with a mass of 2.5 kg, resultant effective area of 11.6 m^2^ (*i.e.*, diameter of 0.496 m), 3 dB bandwidth of 66 kHz, signal-to-noise ratio of 4 (*i.e.*, varying magnetic field strength of 0.2 nT/s), and normalized equivalent input noise of 3.62 nV/m^2^.

All of the parameters of the ACS, such as mass, resultant effective area, 3 dB bandwidth, SNR, and normalized equivalent input noise, can be changed according to the requirements of the mentioned helicopter TEM exploration based on the proposed optimized procedure. Furthermore, to improve the quality of the received signal, the ability of the ACS to suppress the pendulum motion noise caused by the oscillation of the towed bird should be studied in future work.

## Figures and Tables

**Table 1:** Parameters of the two air-core coils.

Parameters	Air-Core Coil A	Air-Core Coil B
Radius	0.152 m	0.248 m
Segments	4	4
Number of turns	160	60
Diameter of wire	0.5 mm	0.5 mm
Resistance of air-core coil	10.46 Ω	3.88 Ω
Inductance of air-core coil	4.05 mH	1.14 mH
Capacitance of air-core coil	773 pF	283 pF
Response frequency	90 kHz	280 kHz

**Table 2:** Components of the pre-amplifier circuit.

Components	Parameters
R_11_ = R_22_	1.3 kΩ
R_4_ = R_6_	1.5 kΩ
R_5_	100 Ω
R_7_ = R_8_	1 kΩ
R_9_ = R_10_	20 kΩ
G	620
U_1_,U_2_	AD797
U_3_	THS4131
